# Capsaicin ameliorates diabetic nephropathy by targeted activation of GPX4 and inhibition of pyroptosis

**DOI:** 10.3389/fendo.2026.1937280

**Published:** 2026-10-07

**Authors:** Congqin Zhang, Xiaorong Wang, Fang Zhang, Chunhui Li, Xue Zhao, Sinan Zhao, Jing Liu, Sumin Jiao, Yongmei Hao

**Affiliations:** 1 Department of Nephrology, The Second Hospital of Hebei Medical University, Shijiazhuang, China; 2 Department of Endocrinology, The Second Hospital of Hebei Medical University, Shijiazhuang, China

**Keywords:** capsaicin, diabetic nephropathy, mitochondrial ultrastructure, NLRP3/caspase-1/GSDMD-N, pyroptosis

## Abstract

**Purpose:**

Capsaicin (CAP) is the primary pungent alkaloid found in chili peppers. The aim of this study is to screen for candidate therapeutic agents targeting the mitochondrial dysfunction–pyroptosis axis in diabetic nephropathy (DN) and to validate the efficacy and mechanism of action of capsaicin.

**Methods:**

Bioinformatics analysis was conducted to identify candidate genes and drugs. The protective effects and underlying mechanisms of CAP were investigated using a high-glucose (HG)-induced HK-2 cell model and a streptozotocin-induced T1DM DN rat model. Experimental techniques included CCK8 assay, flow cytometry (pyroptosis and ROS), JC-1 staining, Western blot analysis, and histopathological staining. GPX4-IN-3 was applied *in vivo* and *in vitro* to determine whether it reversed the protective effects of capsaicin. The data were analyzed using the Shapiro–Wilk test, one-way analysis of variance (ANOVA) with corresponding post-hoc tests, and correlation analysis.

**Results:**

Bioinformatic analysis identified 12 intersecting genes and highlighted CAP as a potential GPX4-targeting compound. Molecular docking demonstrated a high binding affinity of CAP for GPX4 and caspase-9. *In vitro*, CAP dose-dependently restored cell viability and suppressed HG-induced pyroptosis in HK-2 cells. It significantly alleviated mitochondrial damage by reducing ROS production, restoring mitochondrial membrane potential, and improving mitochondrial ultrastructure. Notably, these protective effects were reversed by GPX4-IN-3. Western blot analysis revealed that CAP upregulated GPX4 expression and inhibited activation of the NLRP3/caspase-1/GSDMD-N pyroptosis pathway. *In vivo*, CAP treatment markedly improved renal histopathology and renal function in DN rats; however, these effects were attenuated by co-administration of GPX4-IN-3.

**Conclusion:**

This study demonstrates that CAP ameliorates diabetic renal injury by targeting GPX4, thereby mitigating mitochondrial dysfunction and subsequent NLRP3-mediated pyroptosis. These findings validate the bioinformatic prediction and reveal a novel mechanism of action for CAP, positioning it as a promising therapeutic candidate for DN.

## Introduction

Diabetes mellitus has become a global public health crisis. Among the subtypes of diabetes, type 2 diabetes (T2DM) accounts for the vast majority of cases, with over 537 million adults worldwide suffering from it (1); while type 1 diabetes (T1DM) is a chronic autoimmune disease, although its incidence is relatively low, it is still on the rise (2, 3). Among them, diabetic nephropathy (DN) has become the leading cause of end - stage renal disease (ESRD) globally, posing an increasingly severe public health challenge. The latest data show that the number of diabetes patients worldwide has approached 700 million in 2025 and is expected to exceed 800 million by 2030. Approximately 30% - 40% of diabetes patients will develop diabetic nephropathy, and the incidence rate is increasing at a rate of about 3% per year. The global burden of diabetes - related chronic kidney disease (CKD) continues to grow, and the age - standardized incidence rate shows a significant upward trend (4), highlighting the urgency of seeking new treatment strategies. Recent research has highlighted the critical role of mitochondrial dysfunction in the pathogenesis of DN. Under diabetic conditions, persistent hyperglycemia disrupts mitochondrial homeostasis, leading to impaired oxidative phosphorylation, excessive production of reactive oxygen species (ROS), and, ultimately, cellular damage (5). Therefore, targeting mitochondrial dysfunction is considered a promising strategy to slow the progression of DN.

Mitochondrial damage is not an isolated event; rather, it is closely associated with another key pathological process—pyroptosis (6). Pyroptosis, a proinflammatory form of programmed cell death mediated by gasdermin family proteins, has been identified as a key contributor to renal tubular injury (7). Activation of the NLRP3 inflammasome and subsequent caspase-1-dependent cleavage of the proinflammatory cytokines interleukin-1β (IL-1β) and interleukin-18 creates a vicious cycle of inflammation and cell death (8). Notably, glutathione peroxidase 4 (GPX4), a crucial antioxidant enzyme that counteracts lipid peroxidation, has been recognized as a central regulator linking mitochondrial dysfunction and pyroptosis, thereby presenting a potential therapeutic target for DN intervention (9).

In this context, natural compounds with multitarget potential represent attractive candidates for the management of DN (10). Hu et al. found that berberine improved renal function parameters and lipid metabolism in animals with diabetic nephropathy (11); quercetin alleviated hyperglycemia-induced apoptosis in renal tubular epithelial cells via the PI3K/AKT pathway (12); Resveratrol exerts a nephroprotective effect by regulating SIRT1-mediated podocyte autophagy and inflammatory signaling (13); glutamine alleviated biomarkers of renal inflammation and oxidative stress, thereby reducing diabetic nephropathy and other complications of type 2 diabetes (14); Harmine alleviates DN by regulating the AMPK/Nrf2 pathway and blocking ATM signaling (15). These findings support the therapeutic potential of natural products; however, only a few have been shown to directly target the mitochondrial dysfunction–pyroptosis axis that drives DN progression (16, 17). Capsaicin (CAP), the primary pungent component of chili peppers, has demonstrated diverse pharmacological activities, including antioxidant, anti-inflammatory, and metabolic-modulating effects (18). Epidemiological evidence shows that regular consumption of spicy foods can reduce the risk of type 2 diabetes by 34%. The protective effect is most significant when such foods are consumed 3–5 days a week (19). Previous reports have described an association between CAP and diabetes-related complications (20); however, experimental validation remains limited. Regarding kidney disease, liposome-encapsulated CAP has been reported to effectively alleviate chronic kidney disease (CKD)-associated pruritus, and its effects in CKD involve modulation of excitatory and inhibitory renal reflex pathways (21). Furthermore, CAP is primarily metabolized in the kidneys (22). Although previous studies have suggested potential benefits of CAP in CKD and diabetes, its specific role in DN remains unclear. It should be acknowledged, however, that the pungent property of CAP, mediated through activation of TRPV1 on gastrointestinal sensory nerve fibers, may induce gastric irritation and potentially aggravate gastric mucosal injury in DN patients with active peptic ulcers or inflammatory bowel disease (23); this clinical limitation should be considered in future translational studies and might be addressed by enteric-coated or renal-targeted delivery formulations.

Based on preliminary bioinformatics analysis, CAP was identified as a potential GPX4-targeting compound. Building on these premises, we hypothesized that CAP ameliorates diabetic renal injury by targeting GPX4, thereby restoring mitochondrial homeostasis and suppressing NLRP3/caspase-1/GSDMD-N-mediated pyroptosis. In the present study, a streptozotocin (STZ)-induced type 1 DN rat model was employed, which is characterized by high modeling stability and typical renal pathological phenotypes, serving as a classic *in vivo* model for exploring oxidative stress and cell death mechanisms in DN, with pharmacological reversal using the specific GPX4 inhibitor GPX4-IN-3 and an ACEI as the positive control. These experiments were designed to provide direct experimental evidence supporting CAP as a novel therapeutic candidate for DN.

## Methods

### Data collection and preprocessing

RNA sequencing data from blood samples of healthy volunteers (n = 5) and patients with DN (n = 5) were obtained from the Gene Expression Omnibus dataset GSE154881. Data preprocessing and differential gene expression (DEG) analysis were performed using the DESeq2 R package. A significance threshold of |log2 fold change (log2FC)| > 1 and p < 0.05 was applied to identify genes significantly altered in DN.

### Gene set collection and target screening

A total of 486 pyroptosis-related genes were retrieved from the GeneCards database, 1,315 DN-related genes were obtained from GSE154881, and 1,136 mitochondria-related genes were obtained from the MitoCarta3.0 database. The intersection of these three gene sets was identified for further analysis.

### Network pharmacology analysis

Using DrugBank and the Comparative Toxicogenomics Database, a gene–protein–compound interaction network comprising 43 nodes and 176 edges was constructed based on the intersecting genes identified above. CAP and its interacting proteins encoded by genes within the network were selected for molecular docking analysis.

### Molecular docking

The chemical structure of the active ingredient Capsaicin (PubChem CID: 1548943) was collected in MOL2 format from the TCMSP database (Version 2.3, https://old.tcmsp-e.com/tcmsp.php). The crystal structures of the key targets GPX4 (UniProt: K7ERP4) and Caspase-9 (UniProt: P55211-3) were retrieved from the PDB database (https://www.rcsb.org/). AutoDockTools 1.5.7 was used to remove water molecules, add hydrogens, assign charges, and predict the active binding pocket. Subsequently, AutoDock 4.2 was employed to set the docking box parameters based on the predicted active pocket and perform the docking calculation. Finally, the optimal docking conformation was visualized using PyMOL 3.1.6.1 software.

For the BAX docking system: the grid center is (1.196, 1.106, 1.356) Å; grid points are 54 × 60 × 126; grid spacing is 0.375 Å; box size is 20.25 × 22.50 × 47.25 Å. For the CASP9 docking system: the grid center is (1.196, 1.106, 1.356) Å; grid points are 126 × 126 × 126; grid spacing is 0.375 Å; box size is 47.25 × 47.25 × 47.25 Å. For the GPX4 docking system: the grid center is (−4.314, −0.832, 3.169) Å; grid points are 126 × 126 × 126; grid spacing is 0.26 Å; box size is 32.76 × 32.76 × 32.76 Å. For the PRDX6 docking system: the grid center is (−4.314, −0.832, 3.169) Å; grid points are 126 × 126 × 126; grid spacing is 0.26 Å; box size is 32.76 × 32.76 × 32.76 Å. For the UCP1 docking system: the grid center is (−4.314, −0.832, 3.169) Å; grid points are 126 × 126 × 126; grid spacing is 0.26 Å; box size is 32.76 × 32.76 × 32.76 Å. For all systems, the maximum number of energy evaluations was 2,500,000; the genetic algorithm was run 10 times with a population size of 150; the RMSD clustering tolerance was 2.0 Å; and the random seed was set to pid/time. The final binding conformation was selected based on the lowest predicted binding free energy output by AutoDock; RMSD clustering was used only to identify conformational similarity and did not override the energy-based ranking.

### Cell culture and high-glucose model establishment

Human renal tubular epithelial cells (HK-2, Sunncell, China) were routinely cultured in Dulbecco’s modified Eagle’s medium (SNM-003E, Sunncell, China) supplemented with 10% fetal bovine serum (SNS-002, Sunncell, China) and 1% penicillin–streptomycin in a humidified incubator at 37 °C with 5% CO2 (HF100, Heal Force, China). The cell lines used in this study were recently authenticated by short tandem repeat analysis and were routinely tested for mycoplasma contamination, with negative results. To establish the high-glucose (HG) model, when the cells reached approximately 80% confluence, the normal-glucose (NG; 5.5 mM D-glucose) medium was replaced with high-glucose medium containing 30 mM D-glucose, followed by continued culture for 48 h to induce cellular injury (24).

### *In vitro* cell experiment grouping

To investigate the protective effect of CAP (HY-10448, MedChemExpress, China) and its association with GPX4, the experiment included six groups: NG, HG group, 0.5 μmol/L CAP (L), 1 μmol/L CAP (M), 2 μmol/L CAP (H), and a combination treatment group consisting of CAP (H) and the GPX4 inhibitor GPX4-IN-3 (HY-141809, MedChemExpress, China) (25). Specifically, during HG modeling, CAP at different concentrations (0.5, 1, and 2 μmol/L) was added to the culture medium for intervention. In the combination treatment group, high-dose CAP (2 μmol/L) was co-administered with GPX4-IN-3 (1 μmol/L). All treatments were maintained for 48 h.

### Cell viability assay (CCK-8)

Cell viability was assessed using the Cell Counting Kit-8 (CCK-8) assay. Briefly, HK-2 cells in the logarithmic growth phase were seeded into 96-well plates at a density of 5 × 10^3^ cells per well. After the respective treatments, 10 μL of CCK-8 solution (BA00208, Bioss Biotechnology Co., Ltd., China) was added to each well, followed by incubation at 37 °C in the dark for 2 h. Subsequently, the absorbance was measured at 450 nm using a microplate reader (ELX800, BioTek Instruments, USA). Cell viability was expressed as a percentage relative to the absorbance of the NG group.

### Pyroptosis detection by Annexin V–FITC/PI double staining and flow cytometry

After treatment, cells were collected by trypsinization without ethylenediaminetetraacetic acid and washed twice with precooled phosphate-buffered saline (PBS). Subsequently, according to the manufacturer’s instructions for the fluorescently labeled Annexin V (Annexin V–FITC)/propidium iodide (PI) apoptosis detection kit (40303ES60, Yeasen Biotechnology Co., Ltd., China), the cells were resuspended in 1 × binding buffer, followed by sequential addition of Annexin V–FITC and PI staining solutions, and incubated at room temperature in the dark for 15 min. Detection was performed within 1 h using a flow cytometer (NovoCyte 2070R, Agilent Technologies Inc., China). The cell population positive for both Annexin V–FITC and PI was defined as pyroptotic cells.

### Intracellular ROS level detection by flow cytometry

Intracellular ROS levels were measured using a Reactive Oxygen Species Assay Kit (50101ES01, Yeasen Biotechnology Co., Ltd., China). After treatment, cells were collected, and the DCFH-DA probe was diluted in serum-free medium to a final concentration of 10 μM, followed by resuspension of the cells in the diluted probe solution. The cells were then incubated at 37 °C in the dark for 30 min, with gentle mixing every 5 min to ensure adequate reaction. After incubation, the cells were washed three times with precooled PBS to remove excess probe that had not entered the cells. Finally, the cells were resuspended in PBS, and fluorescence intensity was immediately measured using the FITC channel (excitation/emission = 488/525 nm) of a flow cytometer to reflect intracellular ROS levels.

### Mitochondrial membrane potential detection by JC-1 fluorescent probe

Changes in mitochondrial membrane potential (ΔΨm) were assessed using a JC-1 mitochondrial membrane potential assay kit (717164ES10, Yeasen Biotechnology Co., Ltd., China). Cells from each group were collected, washed with PBS, and incubated with an appropriate volume of JC-1 staining working solution at 37 °C in the dark for 20 min. After incubation, the cells were washed twice with JC-1 staining buffer and immediately observed under a fluorescence microscope (NWTIRF Ultra300, Nanoimaging Biotechnology Co., Ltd., China). Mitochondrial membrane potential was semi-quantitatively evaluated by calculating the ratio of red to green fluorescence intensity (JC-1 aggregates/monomers). A decrease in this ratio indicated a reduction in mitochondrial membrane potential.

### Transmission electron microscopy for observing cell ultrastructure

To observe the typical morphological characteristics of pyroptosis, cells were collected by trypsinization after treatment and fixed overnight at 4 °C in 2.5% glutaraldehyde. The fixed samples were rinsed with PBS, postfixed with 1% osmium tetroxide, dehydrated through a graded ethanol series, and embedded in epoxy resin. Subsequently, 60 nm ultrathin sections were prepared using an ultramicrotome (UC7, Leica Microsystems, China). After double staining with uranyl acetate and lead citrate, cellular ultrastructure was observed and imaged using a transmission electron microscope (HT7800, Hitachi, Japan).

### Animal diabetic nephropathy model establishment

Thirty-six healthy male Sprague–Dawley rats aged 4–6 weeks and weighing 160–180 g, were purchased from Henan Skbeis Biotechnology Co., Ltd. (license number: SCXK [Yu] 2020-0005). The animals were housed at the Experimental Animal Center of the Second Hospital of Hebei Medical University in accordance with institutional guidelines for animal welfare and ethics (Ethics Approval Number: 2025-AE467). After one week of acclimatization, the rats were divided into a control group (n = 6) and a model group (n = 30) using a random number table. Rats in the DN model group were fasted for 12 h and then intraperitoneally injected once daily for 5 consecutive days with freshly prepared streptozotocin (STZ) (HY-13753, MedChemExpress, China) at a dose of 50 mg/kg, dissolved in 0.1 mol/L citrate buffer (pH 4.5). Rats in the control group received an equal volume of citrate buffer. One week after STZ administration, random blood glucose levels were measured from the tail vein using a glucometer. Rats with three consecutive measurements ≥ 16.7 mmol/L were considered to have successfully established the diabetic model (26). Blood glucose concentrations were monitored every two weeks until the experimental endpoint. During this period, no serious adverse reactions requiring the establishment of a compassionate endpoint were observed in any of the rats, and no rats died. At the endpoint, 24-h urine samples were collected using metabolic cages. After centrifugation, the supernatant was collected, and 24-h urinary protein levels were determined using the Coomassie Brilliant Blue method (BC5655, Solarbio, China). Simultaneously, the rats were anesthetized by intraperitoneal injection of 3% sodium pentobarbital (P3761, Sigma-Aldrich, China) at a dose of 40 mg/kg, and blood samples were collected via the abdominal aorta. Serum was separated for measurement of blood glucose (BC2495, Solarbio, China), blood urea nitrogen (S0574M, Beyotime, China), serum creatinine (S0291S, Beyotime, China), cystatin C (E-EL-R0304,Elabscience, China), kidney injury molecule 1 (KIM-1, E-OSEL-R0009, Elabscience, China), neutrophil gelatinase associated lipocalin (NGAL, PN758, Beyotime, China), glutathione peroxidase 4 (GPX4) activity (E-BC-K883-M, Elabscience, China), Glycated Hemoglobin A1c (HbA1c, ELK012, ELK Biotechnology, China) according to the manufacturers’ instructions. After all experiments, the rats were euthanized by an overdose of 3% sodium pentobarbital (120 mg/kg body weight, intraperitoneal injection). All animal experiments were performed in compliance with the ARRIVE guidelines.

### Experimental animal grouping and administration

After successful establishment of the DN model, the rats were randomly divided into five groups (n = 6 per group): DN model group; DN + CAP low-dose group (2.5 mg/kg); DN + CAP high-dose group (10 mg/kg); DN + CAP high-dose (10 mg/kg) + GPX4-IN-3 (5 mg/kg) group; and DN + angiotensin-converting enzyme inhibitor (Perindopril; HY-B0130, MedChemExpress, China) positive control group (4 mg/kg) (27). CAP and GPX4-IN-3 were dissolved in corn oil containing 5% DMSO, while the ACEI was dissolved in saline. Rats in the control group and the DN group were administered 1 mL of corn oil containing 5% DMSO daily; Rats in the CAP low-dose and high-dose groups were administered 1 mL of CAP at the corresponding concentrations daily; rats in the CAP (H) + GPX4-IN-3 group were administered 1 mL of CAP at the corresponding concentrations daily, with an additional 1 mL of GPX4-IN-3 administered on the first day of each week; rats in the DN + ACEI group were administered 1 mL of corn oil containing 5% dimethyl sulfoxide (DMSO) daily, with an additional 1 mL of ACEI administered on the first day of each week. The drug concentration in the solvent was dynamically adjusted based on body weight. All interventions lasted for 9 weeks, and body weight was recorded every two weeks.

### Histopathological specimen preparation

Twenty-four hours after the final administration, all rats were anesthetized by intraperitoneal injection of 3% sodium pentobarbital. After blood collection via the abdominal aorta, both kidneys were rapidly dissected, perfused, and rinsed with precooled physiological saline, blotted dry with filter paper, and weighed. The kidneys were coronally sectioned at the hilum, and portions of the renal cortex were fixed in 4% paraformaldehyde for 48 h. After fixation, tissue blocks were dehydrated through a graded ethanol series, cleared in xylene, infiltrated with paraffin, and embedded. Using a rotary microtome (HS-3315, Jinhua Hisure Scientific Co., Ltd., China), 4 μm-thick consecutive sections were prepared, mounted on glass slides, baked at 60 °C for 2 h, and stored at 4 °C for subsequent use.

### Western blot analysis

Total protein was extracted from cells and tissues of each group using radioimmunoprecipitation assay lysis buffer (PR20001, Proteintech Group, Inc., China) supplemented with protease and phosphatase inhibitors (PR20016, Proteintech Group, Inc., China) on ice. Protein concentration was quantified using the bicinchoninic acid assay (PK10026, Proteintech Group, Inc., China) to determine the loading amount. Equal amounts of protein were separated by sodium dodecyl sulfate–polyacrylamide gel electrophoresis and transferred onto polyvinylidene fluoride membranes. Subsequently, the membranes were blocked with Tris-buffered saline containing Tween 20 (TBST) supplemented with 5% skim milk at room temperature for 1 h. After blocking, the membranes were incubated overnight at 4 °C with the corresponding primary antibodies, including GPX4, NLRP3, caspase-1, cleaved caspase-1, GSDMD-N, caspase-9, cleaved caspase-9, pro-IL-1β, mature-IL-1β and TRPV1. The following day, the membranes were washed three times with TBST and incubated with the appropriate horseradish peroxidase-conjugated secondary antibodies at room temperature for 1 h. After thorough washing with TBST, protein bands were visualized using an enhanced chemiluminescence substrate and detected with a chemiluminescence imaging system. GAPDH was used as the internal control. Band intensities were quantified using ImageJ software. Detailed antibody information and dilution ratios are provided in **Supplementary Table 1**.

### Hematoxylin and eosin staining

Renal tissue paraffin sections were baked at 60 °C for 1 h, followed by deparaffinization in xylene I and II for 15 min each. The sections were then rehydrated through a graded ethanol series (100%, 95%, 85%, and 75%) for 3 min each and rinsed with distilled water. Sections were stained with hematoxylin for 5 min, rinsed with running water, differentiated in 1% acid alcohol for several seconds, and subsequently blued under running water for 15 min. After counterstaining with eosin for 3 min, the sections were dehydrated through a graded ethanol series, cleared in xylene twice for 5 min each, mounted with neutral balsam, and stored at room temperature protected from light. Pathological changes were systematically examined under an optical microscope (XSP-36-1600X, Phenix Optical Company Limited, China) at ×400 magnification. All pathology cases were evaluated by two experts who were unaware of the group assignments.

### Masson trichrome staining

After deparaffinization and rehydration, paraffin sections were processed according to the manufacturer’s instructions for the Masson Trichrome Staining Kit (BTN131272, Beijing Baiao Leibo Technology Co., Ltd., China). Nuclei were stained with Regaud’s hematoxylin for 5 min, rinsed with water, and differentiated. Subsequently, sections were stained successively with Ponceau S–acid fuchsin solution for 5 min, treated with phosphomolybdic acid solution for 5 min, and counterstained with aniline blue solution for 5 min. After staining, sections were treated with 1% glacial acetic acid for 1 min, rapidly dehydrated through 95% ethanol and absolute ethanol, cleared in xylene, and mounted with neutral balsam. Nuclei appeared blue-black, cytoplasm and muscle fibers appeared red, and collagen fibers appeared bright blue. Collagen fiber deposition and blue-stained areas in the glomerular mesangium, renal tubulointerstitium, and blood vessels were observed under an optical microscope, and semi-quantitative analysis was performed using image analysis software.

### Periodic acid–Schiff staining

Renal tissue paraffin sections were routinely deparaffinized and rehydrated, then immersed in 1% periodic acid solution for 10 min and rinsed with distilled water. Subsequently, the sections were incubated with Schiff’s reagent in the dark for 15–20 min, followed by rinsing under running water for 10 min to allow color development. Nuclei were counterstained with hematoxylin for 1–2 min, differentiated in acid alcohol, and blued under running water. After dehydration through a graded ethanol series and clearing in xylene, the sections were mounted with neutral balsam. PAS staining renders glycoproteins in the glomerular basement membrane and mesangial matrix bright magenta, whereas nuclei appear blue. Histopathological changes were systematically examined under an optical microscope.

### Statistical analysis

Each experiment included at least three biological replicates. All data were analyzed using GraphPad Prism 10 software, and results are expressed as mean ± standard deviation (mean ± SD). The Shapiro-Wilk test was used to assess the normality of data within groups, while the Brown-Forsythe test and Bartlett’s test were used to assess the homogeneity of variances between groups. Multigroup comparisons were performed using one-way analysis of variance (ANOVA), followed by Tukey’s *post hoc* multiple comparisons after testing for homogeneity of variances. Correlation analysis was conducted based on the normal distribution characteristics of the data using the Pearson or Spearman methods. A p value < 0.05 was considered statistically significant for all analyses.

## Result

### Identification of potential drugs targeting mitochondrial metabolism in diabetic nephropathy using online databases

Based on the differential gene expression analysis results, the gene list was ranked according to log2FC, and pathway enrichment analysis was performed using GSEA against the Reactome Pathway database. The analysis revealed significant activation of multiple pathways related to mitochondrial translation (e.g., mitochondrial translation initiation and mitochondrial translation termination) in patients with DN, whereas the interferon alpha/beta signaling pathway was markedly suppressed (**Figure 1A**). These findings indicate that mitochondrial dysfunction plays a critical role in DN progression, and that suppression of the interferon alpha/beta signaling pathway may further exacerbate renal inflammatory responses and immune dysregulation.

**Figure 1:**
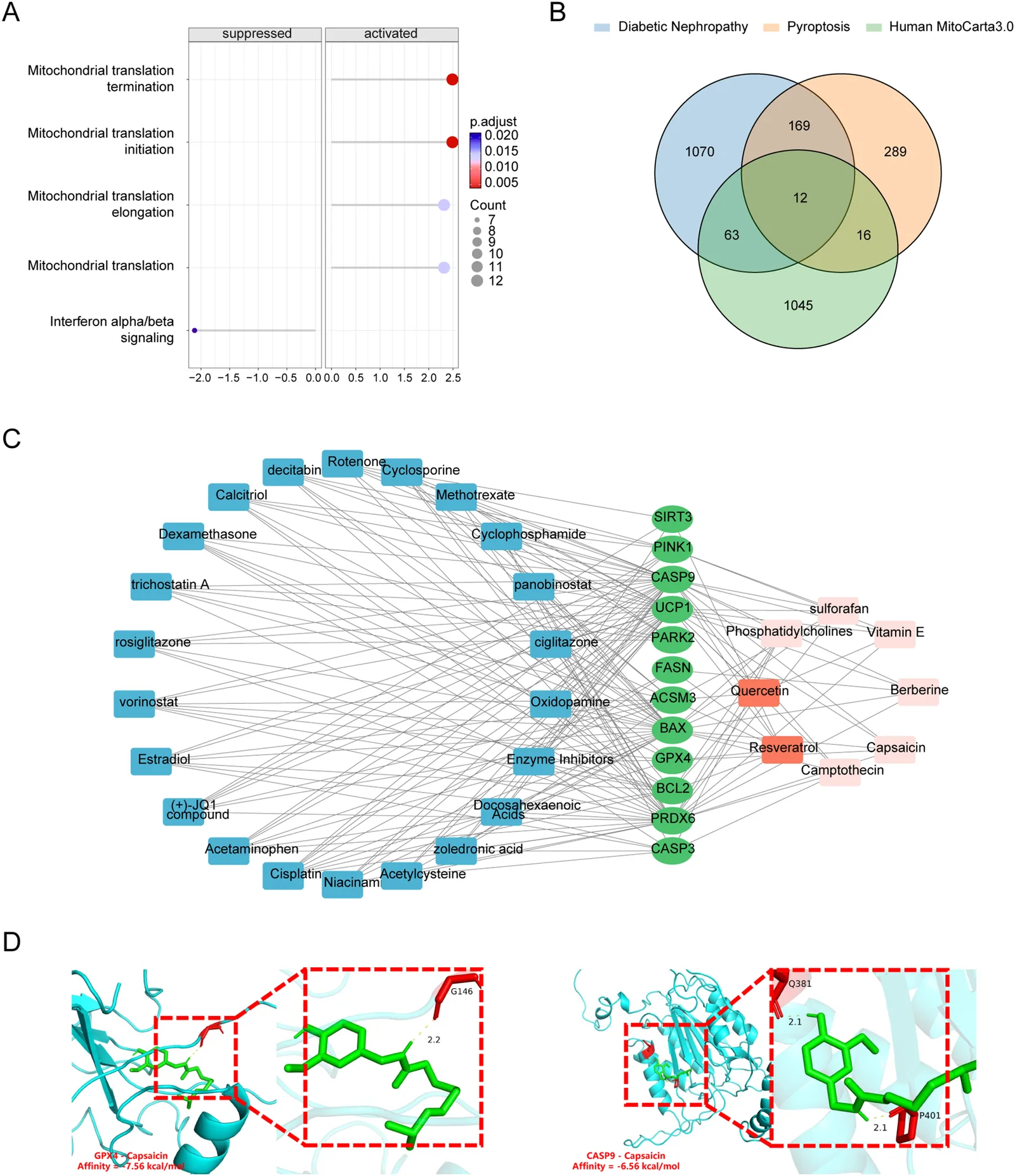
Identification of potential drugs regulating mitochondrial metabolism in diabetic nephropathy based on online databases. **(A)** RNA sequencing data from blood samples of healthy volunteers and patients with DN were obtained from the GSE154881 dataset. GSEA was subsequently performed for pathway enrichment using the Reactome Pathway database. **(B)** A total of 486 pyroptosis-related genes were retrieved from the GeneCards database, along with 1,315 genes associated with diabetic nephropathy. In addition, 1,136 mitochondria-related genes were obtained from the MitoCarta3.0 database. The intersection of these three gene sets is presented as a Venn diagram. **(C)** Using DrugBank and the Comparative Toxicogenomics Database, a gene–protein–compound interaction network comprising 43 nodes and 176 edges was constructed based on the 12 intersecting genes. **(D)** Molecular docking was performed between CAP and GPX4, as well as CASP9.

Subsequently, intersection genes associated with pyroptosis, mitochondrial function, and differentially expressed genes in DN were identified, resulting in 12 key genes: PINK1, ACSM3, BCL2, GPX4, PRKN, CASP9, FASN, PRDX6, CASP3, UCP1, SIRT3, and BAX. These genes are proposed to play important roles in mitochondrial protection and anti-pyroptotic processes (**Figure 1B**). Using DrugBank and the Comparative Toxicogenomics Database, a protein–compound interaction network was constructed based on the proteins encoded by these 12 genes. This network comprised 43 nodes and 176 edges (**Figure 1C**). The topology analysis table of 43 nodes is presented in **Supplementary Table 2**. The network highlighted several plant-derived compounds with known renoprotective effects, such as berberine, quercetin, and resveratrol, and also identified compounds less frequently reported in DN research, including CAP and camptothecin. Camptothecin has been extensively studied and is a common anticancer drug with significant cytotoxicity; therefore, it is not within the scope of this study. Molecular docking analysis was performed between CAP and five interacting proteins (BAX, CASP9, GPX4, PRDX6, and UCP1) encoded by these genes. The docking results demonstrated that CAP exhibited high binding affinity for GPX4 and CASP9, with binding energies of −7.56 kcal/mol and −6.56 kcal/mol, respectively (**Figure 1D**). Topological analysis shows that CASP9 has a degree of 30 and a betweenness centrality of 0.1900, making it, along with BAX, the most central hub node in the network. As an interacting protein of CAP, CASP9 exerts a high level of network influence at the systems level. Although GPX4’s topological metrics (degree = 10) are lower than those of CASP9, molecular docking results show that the binding energy between CAP and GPX4 is −7.56 kcal/mol, representing the highest affinity among all predicted targets and significantly exceeding that of the CAP–CASP9 interaction (**Supplementary Table 2**).

### CAP restores proliferation and suppresses pyroptosis in HK-2 cells under high-glucose conditions

To investigate the effects of CAP on HK-2 cell proliferation and pyroptosis, an *in vitro* HG model was established using HK-2 cells. To determine whether CAP exerts its effects by targeting GPX4, the GPX4 inhibitor GPX4-IN-3 was introduced for reverse validation. CCK-8 assay results demonstrated that treatment with various concentrations of CAP for 48 h did not induce cytotoxicity in HK-2 cells (**Figure 2A**). Under high-glucose conditions, cell viability in the HG group was significantly lower than that in the NG group. CAP treatment significantly increased cell viability in a concentration-dependent manner (**Figure 2B**). We selected 0.5, 1, and 2 μmol/L as the low, medium and high treatment groups for CAP. Flow cytometric analysis revealed that the pyroptosis rate was significantly higher in the HG group than in the NG group. CAP treatment significantly reduced the pyroptosis rate compared with the HG group, with the high-dose CAP group exhibiting the most pronounced effect. Co-treatment with GPX4-IN-3 markedly reversed the protective effect of high-dose CAP, resulting in a significant increase in the pyroptosis rate (**Figure 2C**). These findings indicate that CAP alleviates high-glucose-induced suppression of cell proliferation and inhibits pyroptosis, likely through modulation of GPX4.

**Figure 2:**
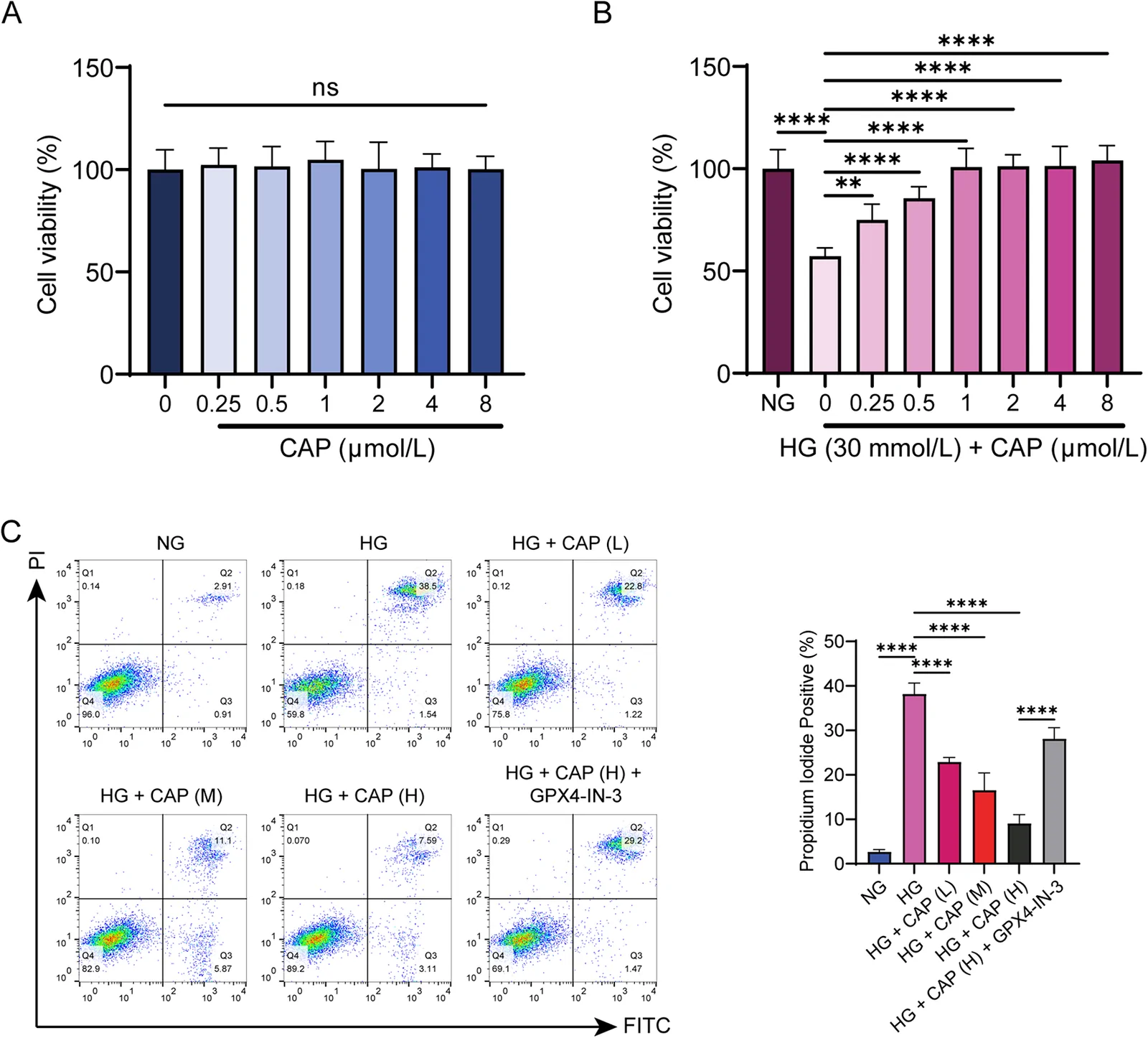
CAP restores proliferation and inhibits Ppyroptosis of HK-2 cells in a high-glucose model. **(A)** CCK8 assay was used to assess cell viability in HK-2 cells treated with different concentrations of CAP (0.25, 0.5, 1, 2, 4, and 8 μmol/L) for 48 h (n = 6). **(B)** Under high-glucose conditions (30 mmol/L glucose), the CCK-8 assay was performed to evaluate cell viability in HK-2 cells treated with various concentrations of CAP (0.25, 0.5, 1, 2, 4, and 8 μmol/L) for 48 h (n = 6). **(C)** Under high-glucose conditions, HK-2 cells were treated with different concentrations of CAP (0.5, 1, and 2 μmol/L) in the presence or absence of 1 μmol/L GPX4-IN-3. Flow cytometry using FITC/PI double staining was performed to evaluate pyroptosis in HK-2 cells (n = 5). “ns” indicates no statistical significance (p > 0.05). **p < 0.01; ****p < 0.0001.

### CAP inhibits high-glucose-induced pyroptosis of HK-2 cells by alleviating mitochondrial damage

The effects of CAP on mitochondrial function under high-glucose conditions were further investigated. Flow cytometric analysis demonstrated that intracellular ROS levels were significantly elevated under HG conditions. CAP treatment significantly reduced ROS levels in a concentration-dependent manner, with the high-dose CAP group showing the most pronounced effect. This reduction was reversed by co-treatment with GPX4-IN-3, resulting in a significant rebound in ROS levels (**Figure 3A**). Changes in ΔΨm were subsequently assessed. ΔΨm was significantly decreased under HG conditions. CAP treatment significantly restored ΔΨm, whereas this effect was abolished by the addition of GPX4-IN-3 (**Figure 3B**). TEM revealed marked cellular swelling and an increased number of balloon-like pyroptotic bodies in the HG group. CAP treatment ameliorated these morphological alterations, and this protective effect was negated by GPX4-IN-3 co-treatment (**Figure 3C**). These findings indicate that HG conditions impair mitochondrial structure and function, and that CAP significantly mitigates this damage, likely through modulation of GPX4.

**Figure 3:**
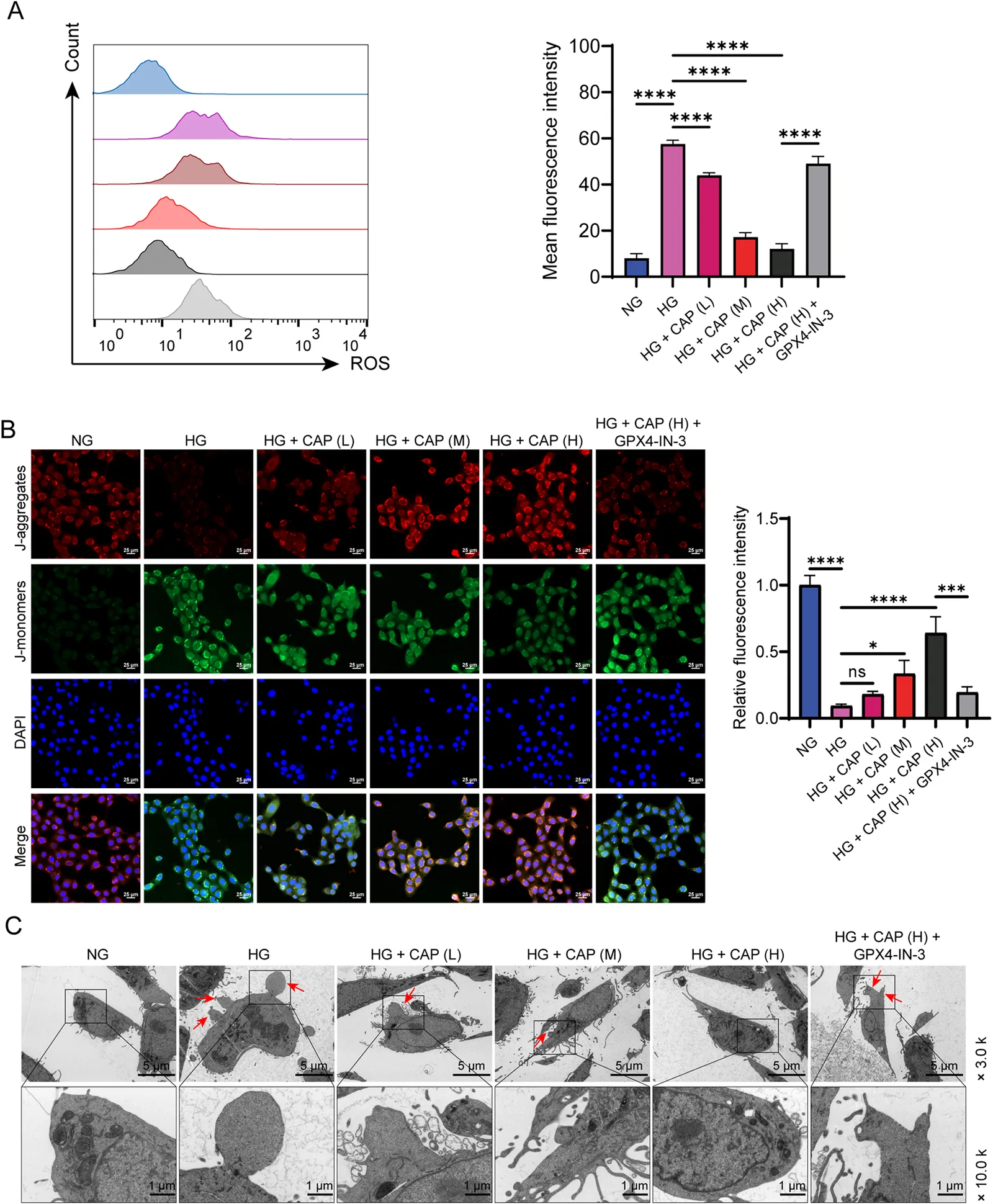
CAP inhibits high - glucose - induced pyroptosis of HK - 2 cells by alleviating mitochondrial damage. **(A)** To investigate the effects of CAP on intracellular ROS levels in HK-2 cells under high-glucose conditions, cells were treated with CAP or GPX4-IN-3. Intracellular ROS levels were measured by flow cytometry (n = 5). **(B)** Mitochondrial membrane potential in HK-2 cells from each group was assessed using the JC-1 fluorescent probe (×400; scale bar: 25 μm; n = 3). **(C)** Cellular ultrastructure was examined by transmission electron microscopy (×3,000; scale bar: 5 μm; ×10,000; scale bar: 1 μm; n = 3). Red arrows indicate pyroptotic cells exhibiting balloon-like morphology and pyroptotic bodies. “ns” indicates no statistical significance (p > 0.05). *p < 0.05; **p < 0.01; ***p < 0.001; ****p < 0.0001.

### CAP inhibits high-glucose-induced pyroptosis of HK-2 cells through activation of GPX4 and regulation of pyroptosis-related signaling pathways

To elucidate the underlying mechanism of CAP, Western blot analysis was performed to examine changes in key pyroptosis-related proteins and signaling pathways. Compared with the NG group, the HG group exhibited significantly increased ratios of cleaved caspase-1/caspase-1 and cleaved caspase-9/caspase-9, significantly decreased GPX4 protein expression, and significantly elevated protein levels of GSDMD-N, pro-IL-1β, mature-IL-1β, and NLRP3. Treatment with different concentrations of CAP reversed these alterations in protein expression. The addition of GPX4-IN-3 counteracted the effects of CAP (**Figure 4A**). These findings indicate that CAP attenuates HG-induced pyroptosis in HK-2 cells through activation of GPX4 and inhibition of the NLRP3/IL-1β signaling pathway.

**Figure 4:**
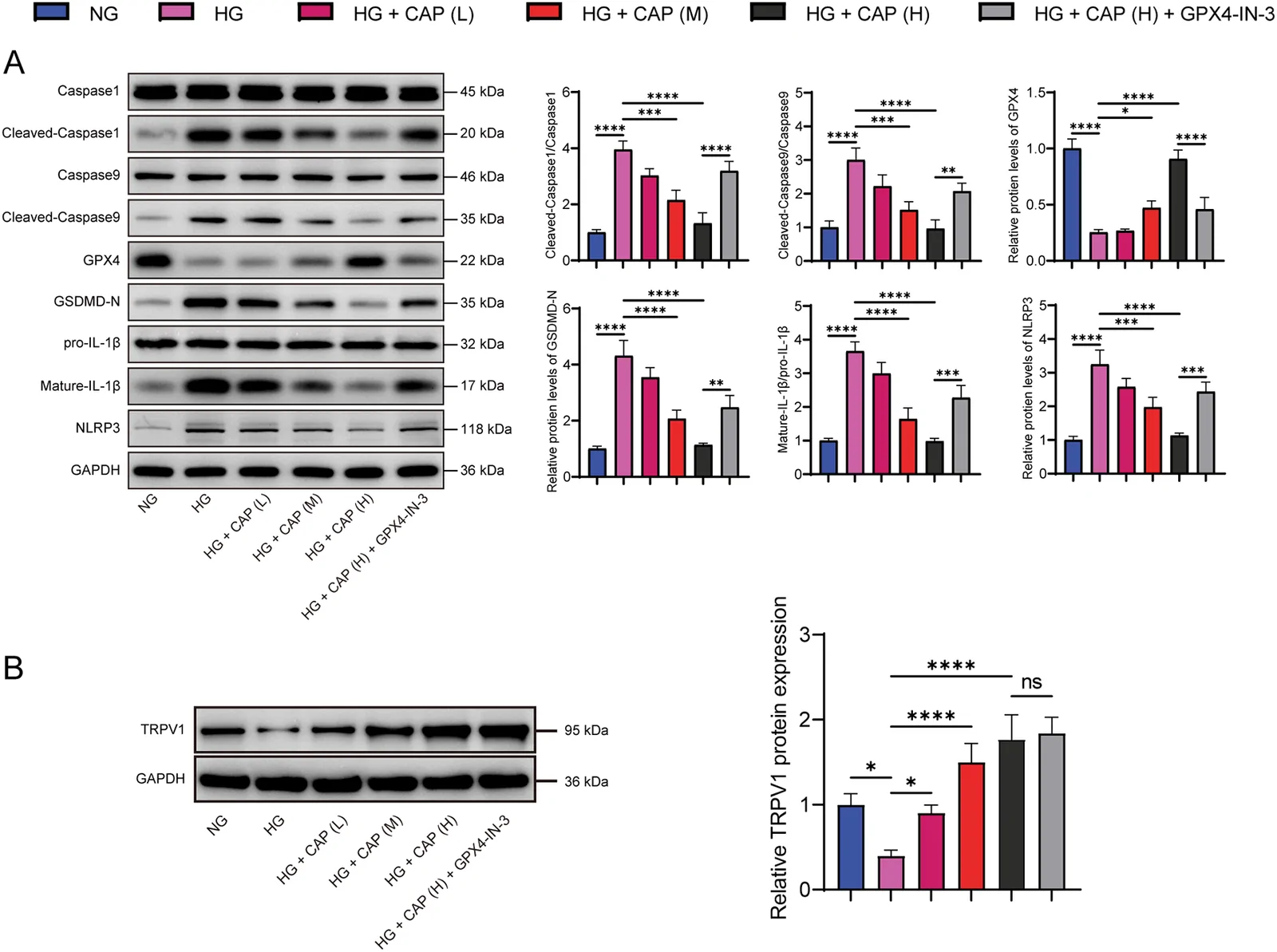
CAP inhibits High-Glucose-Induced Pyroptosis of HK-2 cells through activation of GPX4 and regulation of pyroptosis-related signaling pathways. **(A)** To elucidate the molecular mechanism of CAP, HK-2 cells cultured under high-glucose conditions were treated with different concentrations of CAP or GPX4-IN-3. The expression levels of key proteins involved in pyroptosis were detected by Western blot, including caspase-1, cleaved caspase-1, caspase-9, cleaved caspase-9, GPX4, GSDMD - N, pro-IL-1β, mature-IL-1β, and NLRP3 (n = 3). **(B)** Representative Western blot bands for TRPV1 protein in HK-2 cells in each group and quantification of relative expression (n = 3). “ns” indicates no statistical significance (p > 0.05). *p < 0.05; **p < 0.01; ***p < 0.001; ****p < 0.0001.

CAP is a TRPV1 agonist; activation of TRPV1 can improve muscle atrophy and regulate renal hemodynamics and excretory function, among other effects (28, 29). TRPV1 can also alleviate DN-induced mitochondrial dysfunction in renal podocytes (30). Therefore, this study conducted a brief evaluation of the TRPV1 signaling pathway. Western blot (WB) results showed that, compared with the control group, TRPV1 protein expression was significantly reduced in cells from the HG group. TRPV1 expression recovered following CAP dose-dependent intervention, with the CAP (H) group showing significantly higher levels than the HG group and the HG+CAP (L) group, indicating that CAP effectively activates the TRPV1 signaling pathway in renal epithelial cells. Notably, when GPX4-IN-3 was added to the HG+CAP(H) group, there was no significant change in TRPV1 expression levels, indicating that the GPX4 inhibitor does not affect the activation of TRPV1 by CAP (**Figure 4B**).

### CAP improves renal histopathological damage in a diabetic nephropathy rat model

To comprehensively evaluate the therapeutic efficacy of CAP in DN, relevant biochemical parameters were systematically assessed. During the experiment, the body weight of rats in the control group continued to increase, reaching approximately 500 g by week 9, while their blood glucose levels remained stable at 6 mmol/L; in the DN group, body weight gain slowed significantly after week 2, and blood glucose levels remained consistently above 20 mmol/L. HbA1c levels were also significantly higher than those in the control group. CAP(H) and the positive control drug ACEI were more effective at restoring blood glucose levels. Following the combined administration of GPX4-IN-3, these improvements were significantly reversed (**Figures 5A–C**).

**Figure 5:**
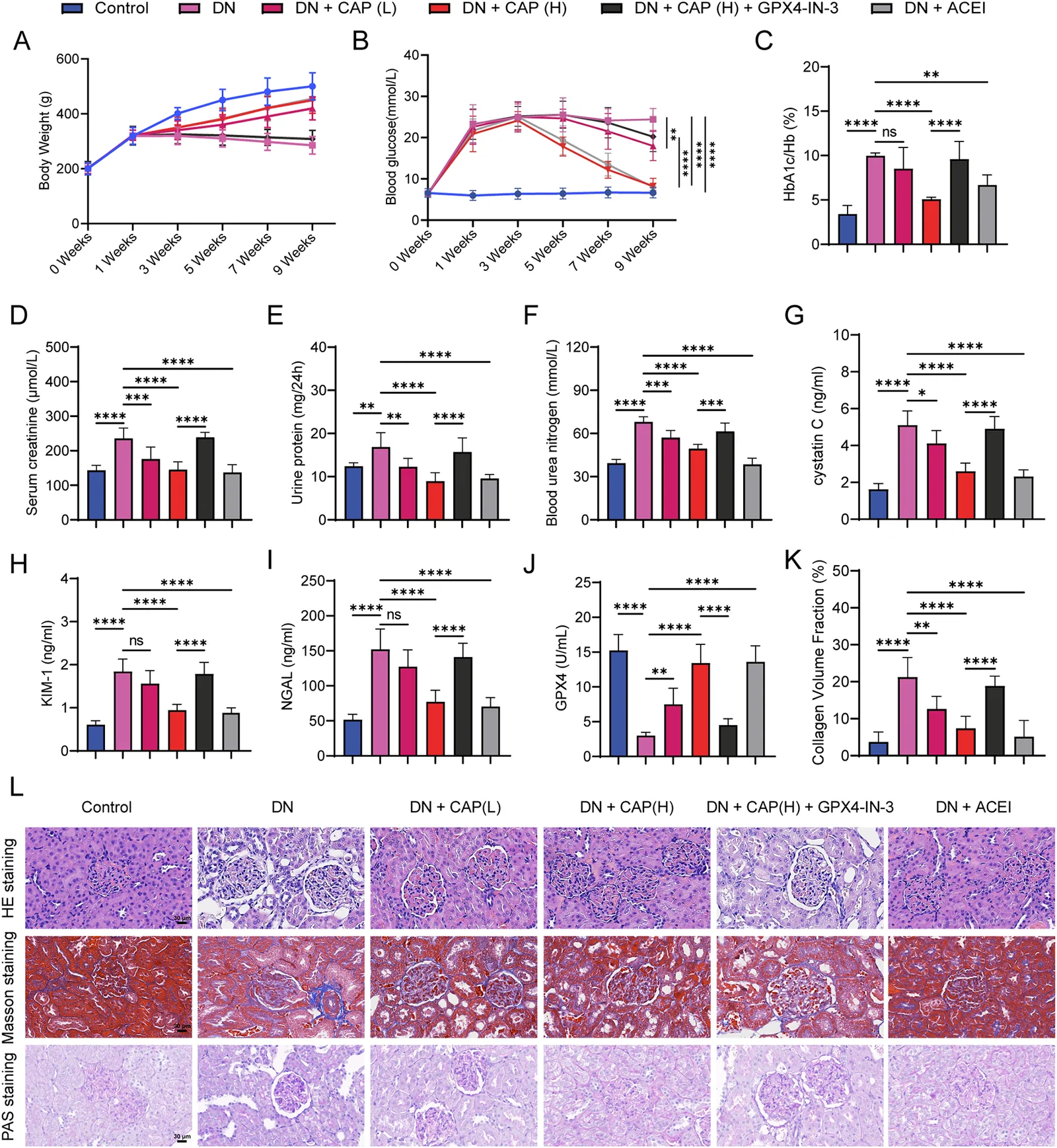
CAP improves renal function and reduces histopathological damage in rats with diabetic nephropathy (DN). In the DN rat model, rats were treated with different doses of CAP (2.5 and 10 mg/kg), 4 mg/kg of ACEI, or a combination of CAP (10 mg/kg) and the GPX4 inhibitor GPX4-IN-3 (5 mg/kg). **(A)** Body weight changes over the 9-week intervention in each group (n = 6). **(B)** Changes in random blood glucose levels of STZ-induced diabetic rats over time (n = 6). **(C–J)** Biochemical indicators related to kidney function and markers of kidney injury: HbA1c **(C)**, serum creatinine **(D)**, 24-hour urine protein **(E)**, blood urea nitrogen **(F)**, cystatin C **(G)**, KIM-1 **(H)**, NGAL **(I)**, and GPX4 activity **(J)** in serum (n = 6). **(K)** The collagen volume fraction (CVF) in Masson-stained sections was quantified using ImageJ software. (n = 6). **(L)** morphological changes in the glomeruli and tubulointerstitium were evaluated by H&E staining (×400; scale bar: 30 μm; n = 6). Masson trichrome staining was used to assess renal interstitial collagen deposition (×400; scale bar: 30 μm; n = 6). PAS staining was performed to evaluate alterations in the mesangial matrix and glomerular basement membrane (×400; scale bar: 30 μm; n = 6). “ns” indicates no statistical significance (p > 0.05). **p < 0.01; ***p < 0.001; ****p < 0.0001.

Compared with the control group, the DN model group exhibited significantly elevated levels of serum creatinine (**Figure 5D**), 24-h urinary protein (**Figure 5E**), blood urea nitrogen (**Figure 5F**), cystatin C (**Figure 5G**), KIM-1 (**Figure 5H**), and NGAL (**Figure 5I**), indicating successful establishment of the DN model accompanied by hyperglycemia and marked renal dysfunction. At the same time, GPX4 activity in serum from the DN group was also significantly reduced (**Figure 5J**). Following treatment with different doses of CAP, these parameters showed dose-dependent improvement. However, co-administration of the GPX4 inhibitor GPX4-IN-3 significantly reversed the beneficial effects of high-dose CAP on these biochemical markers. Furthermore, the positive control ACEI group also showed significant improvement in DN-associated metabolic abnormalities and renal dysfunction, consistent with its established pharmacological effects.

To evaluate the effects of CAP on renal histopathological injury in a DN rat model, H&E, Masson trichrome, and PAS staining were performed. Histological assessment demonstrated that, compared with the control group, the DN model group exhibited marked pathological injury characterized by glomerulosclerosis, aggravated renal tubulointerstitial fibrosis, and thickening of the glomerular basement membrane (**Figure 5L**). In addition, there was a significant increase in blue-stained collagen fibers in the renal interstitium and periglomerular regions of the rats (**Figure 5K**). Following CAP intervention at different doses, these pathological changes were significantly ameliorated. The CAP low-dose group showed partial improvement, whereas the CAP high-dose group exhibited more pronounced restoration of glomerular architecture and reduction of tubulointerstitial damage. However, co-administration of the GPX4 inhibitor GPX4-IN-3 substantially attenuated the renoprotective effects of high-dose CAP, thereby reversing its therapeutic benefits. As a positive control, the ACEI treatment group also demonstrated significant amelioration of renal injury in the DN model.

### CAP inhibits pyroptosis in the renal tissue of diabetic nephropathy model rats through activation of GPX4

Changes in pyroptosis-related proteins and signaling pathways were subsequently examined in rat renal tissues. Compared with the control group, the DN model group exhibited significantly increased ratios of cleaved caspase-1/caspase-1 and cleaved caspase-9/caspase-9, significantly decreased GPX4 protein expression, and significantly elevated levels of GSDMD-N, pro-IL-1β, mature-IL-1β, and NLRP3. Administration of CAP at different doses, as well as ACEI, reversed these aberrant protein expression patterns. Co-administration of GPX4-IN-3 counteracted the effects of CAP (**Figure 6A**). The results of GPX4 immunohistochemistry were consistent with those of Western blot analysis: the area of GPX4-positive staining in renal tissue from the DN group was significantly lower than that in the control group; following CAP or ACEI intervention, the proportion of GPX4-positive area increased in a dose-dependent manner; and after combination with GPX4-IN-3, the proportion of GPX4-positive area decreased again (**Figures 6B, C**). We further evaluated the TRPV1 signaling pathway. TRPV1 protein expression was significantly reduced in renal tissue from the DN group; it recovered following CAP intervention. However, after combination with GPX4-IN-3, TRPV1 expression levels did not show significant changes, consistent with the trends observed in cellular experiments (**Figure 6D**). This also suggests that CAP may activate the TRPV1 receptor, upregulate GPX4 expression, and ultimately inhibit pyroptosis in renal tissue cells (31).

**Figure 6:**
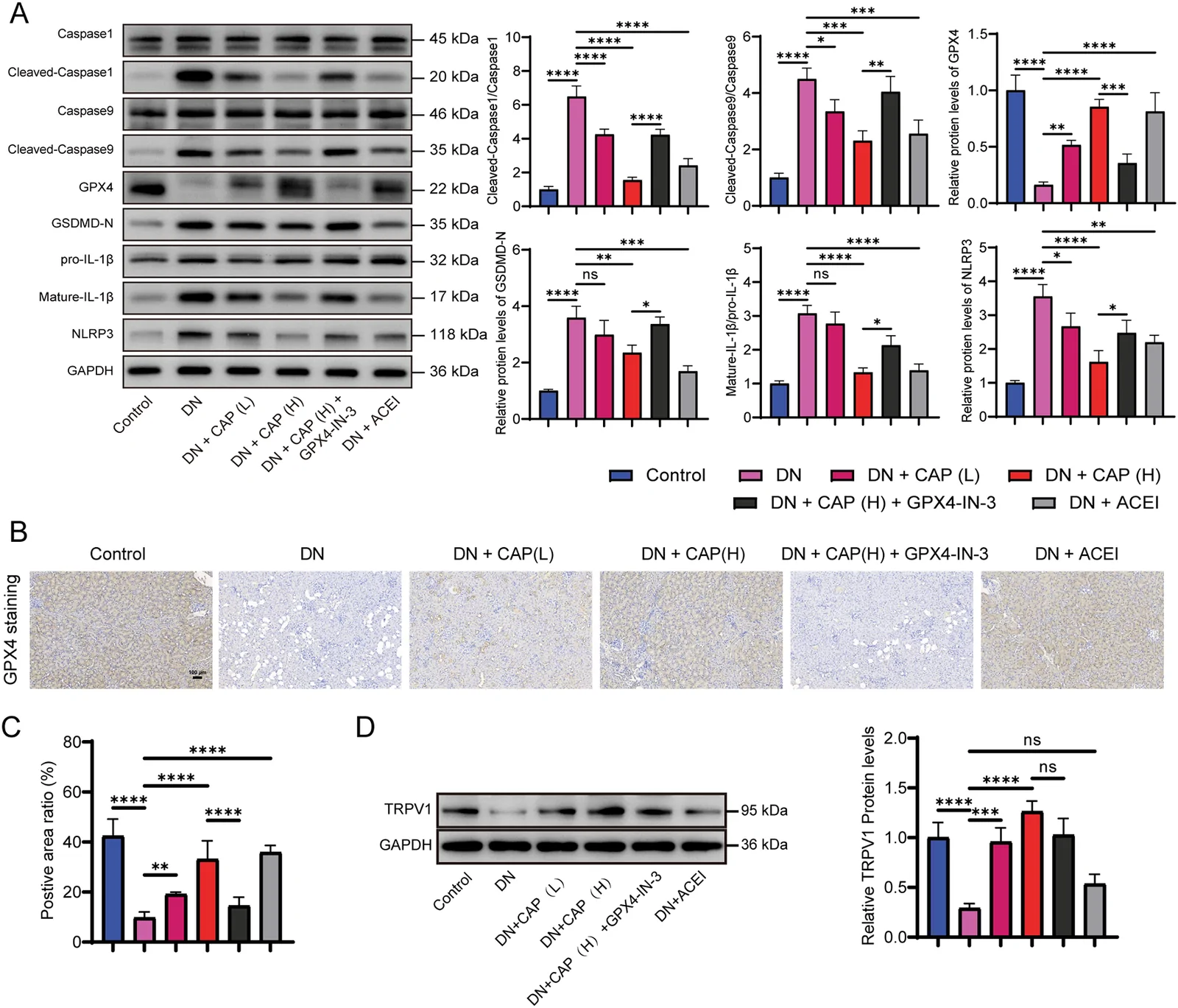
CAP inhibits pyroptosis in the renal tissue of diabetic nephropathy (DN) model rats through activation of GPX4. **(A)** The expression levels of key proteins involved in the pyroptosis signaling pathway in renal tissue were detected by Western blot (n = 3). The analyzed proteins included GPX4, NLRP3, caspase-1, cleaved caspase-1, caspase-9, cleaved caspase-9, GSDMD-N, pro-IL-1β, and mature-IL-1β. **(B)** Representative GPX4 immunohistochemical staining images of renal tissue from rat in each group (×200; scale bar: 50 μm; n = 6). **(C)** Quantification of the percentage of GPX4-positive area (n = 6). **(D)** Representative Western blot bands for TRPV1 protein in renal tissue from rats in each group and quantification of relative expression (n = 3). “ns” indicates no statistical significance (p > 0.05). *p < 0.05; **p < 0.01; ***p < 0.001; ****p < 0.0001.

To clarify the quantitative relationship between GPX4 expression levels and renal dysfunction as well as the degree of pyroptosis activation, we performed Pearson correlation analyses using the relative GPX4 protein expression levels in renal tissue from rats in each group as the independent variable, and the corresponding renal function parameters and levels of pyroptosis-related proteins in each individual as the dependent variables (**Supplementary Figure S1**). The results showed that GPX4 expression was negatively correlated with serum creatinine (r = −0.9330, P < 0.0001), BUN (r = −0.9155, P < 0.0001), 24-hour urinary protein (r = −0.7731, P = 0.0002), serum cystatin C (r = −0.9097, P < 0.0001), KIM-1 (r = −0.8763, P < 0.0001), NGAL (r = −0.8190, P < 0.0001), and HbA1c (r = −0.7008, P = 0.0012); At the same time, GPX4 expression was significantly negatively correlated with Cleaved-Caspase-1/Caspase-1 (r = −0.9149, P < 0.0001), Cleaved-Caspase-9/Caspase-9 (r = −0.8909, P < 0.0001), GSDMD-N (r = −0.8661, P < 0.0001), NLRP3 (r = −0.8261, P < 0.0001), and Mature-IL-1β (r = −0.8896, P < 0.0001)—markers of pyroptosis activation—in renal tissue. These results confirm that higher levels of GPX4 expression in renal tissue are associated with milder renal dysfunction, metabolic disturbances, and pyroptosis activation, further supporting the mechanism by which CAP inhibits renal pyroptosis and improves renal function in DN by upregulating GPX4.

## Discussion

This study employed bioinformatic analysis to identify differentially expressed genes in DN. These genes were subsequently intersected with genes associated with pyroptosis and mitochondrial function to generate a focused gene set for further investigation. Among the candidate gene-associated compounds, CAP and camptothecin were identified as having relatively limited prior investigation in the context of DN. However, research on camptothecin is primarily concentrated in oncology, and its considerable toxicity limits its suitability for DN-related studies (32). In contrast, CAP has been investigated in diabetes and related chronic kidney diseases (20), supporting its potential relevance. Therefore, CAP was selected as the primary candidate for subsequent experimental validation.

In the *in vitro* HG model, CAP dose-dependently restored HK-2 cell viability and significantly suppressed high-glucose-induced pyroptosis. Notably, the GPX4 inhibitor GPX4-IN-3 reversed the protective effects of CAP, suggesting that CAP exerts its renoprotective effects, at least in part, through modulation of GPX4. These results suggest that CAP has an extremely high binding affinity for GPX4 and caspase-9. The reversal experiment with a GPX4 inhibitor further validates this prediction, providing a mechanistic basis for the renoprotective effect of CAP: CAP protects renal tubular cells from high - glucose damage by directly targeting GPX4 to enhance antioxidant defense and simultaneously inhibiting caspase - 9 - mediated death signals. These results are consistent with the reported renoprotective effects of other compounds identified through the bioinformatic screening for DN, including berberine (11), quercetin (12), resveratrol (33), and sulforaphane (34). Moreover, the bioinformatic analysis demonstrated that these compounds share overlapping targets with CAP, providing additional support for the proposed mechanism. Our findings are consistent with and expand upon previous studies on the effects of TRPV1 agonists in diabetic nephropathy (DN). Wei et al. reported that dietary capsaicin improved renal structural and functional damage in db/db and STZ-induced diabetic mice in a TRPV1-dependent manner, acting through the AMPK–FUNDC1 signaling pathway to inhibit endoplasmic reticulum–mitochondrial contact and mitochondrial Ca^2+^ overload in podocytes (30). Our data are similar to those reported but reveal an additional innovative pathway; CAP likely exerts its effects via TRPV1-GPX4. However, while Wei et al. focused primarily on podocytes and the endoplasmic reticulum–mitochondrial contact–AMPK axis, we focused more on renal tubular epithelial cells (HK-2) exposed to high glucose and rat kidneys, highlighting CAP’s protective effects against kidney injury and identifying new targets of action.

Pyroptosis, a form of programmed inflammatory cell death, is closely associated with mitochondrial dysfunction. Under stimuli such as oxidative stress, mitochondrial membrane lipids undergo peroxidation, a process negatively regulated by the key antioxidant enzyme GPX4 (35). Inactivation or depletion of GPX4 leads to the accumulation of lipid peroxides (e.g., malondialdehyde), disrupts mitochondrial membrane integrity, and triggers ΔΨm collapse and ROS overproduction, thereby constituting an early event in mitochondrial damage (36). The present study demonstrates that CAP significantly upregulates GPX4 expression, reduces ROS levels, and restores mitochondrial membrane potential, thereby alleviating mitochondrial structural damage, as further supported by transmission electron microscopy findings. This evidence also positions GPX4 at the intersection of ferroptosis and pyroptosis, as GPX4’s role in ferroptosis is well established (37). GPX4 not only regulates ferroptosis but also acts as an upstream signal to initiate the assembly of the NLRP3 inflammasome. Bai et al. found that sufficient levels of GPX4 inhibit the NLRP3/caspase-1 axis and attenuate ketamine-induced damage to the rat hippocampus (38); miR-1656 can activate the NLRP3 inflammasome by targeting GPX4, leading to the release of the inflammatory cytokines IL-1β and IL-18 and inducing pyroptosis in selenium-deficient chicken kidney tissue (39). Regarding the pyroptosis pathway, damage-associated signals released from dysfunctional mitochondria (e.g., ROS and mitochondrial DNA) are recognized by the cytoplasmic pattern recognition receptor NLRP3, which subsequently assembles with the adaptor protein apoptosis-associated speck-like protein containing a CARD and pro-caspase-1 to form the NLRP3 inflammasome (40). The activated inflammasome cleaves and activates caspase-1, generating cleaved caspase-1. Cleaved caspase-1 exerts dual functions: it cleaves pro-IL-1β into mature-IL-1β, and it cleaves gasdermin D (GSDMD) to generate its N-terminal fragment (GSDMD-N). GSDMD-N forms pores in the plasma membrane, ultimately leading to osmotic cell lysis and release of inflammatory mediators, thereby executing pyroptosis (41). The findings of this study demonstrate that CAP effectively inhibits activation of the NLRP3/caspase-1 signaling pathway. In summary, CAP upregulates GPX4 to maintain mitochondrial homeostasis while concurrently suppressing both the NLRP3/caspase-1-mediated pyroptosis pathway and the caspase-9-mediated apoptotic pathway, thereby exerting multifaceted cytoprotective effects.

## Limitations

Although this study demonstrates through *in vitro* and *in vivo* experiments that CAP ameliorates diabetic kidney injury by targeting GPX4 to regulate mitochondrial function and pyroptosis, several limitations should be acknowledged. First, the STZ-induced diabetic nephropathy model used in this study should be classified as a typical pathological phenotype of T1DM DN. It primarily induces persistent hyperglycemia by chemically and specifically destroying pancreatic β-cells, thereby consistently reproducing typical phenotypes (42, 43). Crucially, however, the molecular characteristics observed in the renal tissue of this model exhibit a high degree of homology with the core pathological pathways underlying the onset and progression of human DN. This model can realistically simulate the damage patterns and molecular regulatory mechanisms of renal parenchymal cells during the progression of DN and is one of the models widely used in numerous publications. At the same time, it must be objectively acknowledged that Type 1 and Type 2 DN still exhibit certain differences in etiology, systemic metabolic profiles, and patterns of renal damage progression. The pharmacodynamic evaluations and mechanistic conclusions obtained in this study based on the T1DM DN model still require further validation in animal models that more closely mimic the pathological characteristics of human T2DM DN. Future studies will employ the db/db spontaneous T2DM model to more systematically evaluate the mechanisms of CAP. Second, the network pharmacology analysis predicts that CAP may interact with multiple targets, such as CASP9. However, the subsequent experimental validation primarily focused on GPX4, and the direct effects of CAP on other potential targets remain unclear. As a transient receptor potential vanilloid 1 (TRPV1) agonist, it remains to be determined whether the renal protective effects of CAP act through the GPX4-TRPV1 signaling pathway. Third, tissue samples were collected uniformly at the end of the intervention, and the effectiveness of the DN model was assessed based on urinary protein and a series of serum markers. Renal function should also be evaluated by directly measuring creatinine clearance and GFR *in vivo*. Future studies should employ metabolic cages to more accurately measure these key indicators. Fourth, although the oral doses of CAP (2.5 and 10 mg/kg) were selected based on the dose range reported in previous studies using metabolic disease models, this study did not conduct specific pharmacokinetic or dose-response analyses in rats with diabetic nephropathy (DN). The optimal therapeutic window, bioavailability, and renal exposure of CAP remain to be determined. At the same time, for GPX4-IN-3 and ACEI, a more frequent dosing regimen would be more ideal from a methodological standpoint, as it would provide a good comparison to the CAP dosing regimen. However, due to limitations in our own conditions, although we preliminarily demonstrated the efficacy of these two groups in this paper, a daily dosing regimen would still be more robust for future studies and could also relatively shorten the rearing period. Fifth, all experiments were conducted in rodent models and *in vitro* cultures of human renal tubular cells; validation in human kidney tissue or clinical trials is required before drawing translational medical conclusions.

## Future perspectives

This study identifies several directions for future research. Future studies could focus on systematic dose optimization and long-term safety/toxicity assessments; given CAP’s pungent properties, its gastrointestinal tolerability should also be monitored. TRPV1 dependence could be directly investigated using TRPV1 antagonists or kidney-specific TRPV1 knockout mice, and the upstream mechanisms by which CAP regulates GPX4 could be elucidated; The efficacy of combination therapy with CAP and established nephroprotective drugs (such as ACEIs or SGLT2 inhibitors) could be explored, as well as the development of enteric-coated or kidney-targeted formulations for patients with diabetic nephropathy (DN) who have peptic ulcers.

## Conclusion

This study demonstrates that CAP ameliorates diabetic nephropathy by upregulating GPX4, restoring mitochondrial homeostasis, and inhibiting NLRP3/caspase-1/GSDMD-N-mediated pyroptosis. Reversal of these renoprotective effects by the GPX4 inhibitor GPX4-IN-3 both *in vitro* and *in vivo* confirms GPX4 as the key mechanistic mediator. These findings validate our bioinformatic prediction and uncover a novel pharmacological mechanism linking mitochondrial dysfunction to inflammatory cell death in the diabetic kidney. Given its multitarget properties, CAP emerges as a promising therapeutic candidate for diabetic nephropathy.

## Data Availability

The original contributions presented in the study are included in the article/**Supplementary Material**, further inquiries can be directed to the corresponding author/s.

## Supplementary material

Supplementary Table 1 Antibody information used in this study.

Supplementary Table 2 Network Topology.

Supplementary Figure 1 Analysis of the Correlation Between GPX4 Expression Levels and Renal Function, Kidney Injury, and the Degree of Pyroptosis Activation Western blot.
