# Supplementary material for: Capsaicin ameliorates diabetic nephropathy by targeted activation of GPX4 and inhibition of pyroptosis
Source: Front Endocrinol (Lausanne). 2026 Oct 7;17:1937280. doi: 10.3389/fendo.2026.1937280 (PMC13683011; doi:10.3389/fendo.2026.1937280)
Supplement: Supplementary file 1 [file Table1.docx]

**Supplementary Table 1 Antibody information used in this study**

| Gene name | Manufacturer | Article number | Dilution ratio |
| --- | --- | --- | --- |
| Caspase-1 | Affinity biosciences | AF5418 | 1: 1000 |
| Cleaved-caspase-1 | Affinity biosciences | AF4005 | 1: 1000 |
| Caspase-9 | Affinity biosciences | AF6348 | 1: 1000 |
| Cleaved-caspase-9 | Affinity biosciences | AF5240 | 1: 1000 |
| GPX4 | Affinity biosciences | DF6701 | 1: 1000 |
| GSDMD-N | Affinity biosciences | DF13758 | 1: 1000 |
| pro-IL-1β | Affinity biosciences | AF5103 | 1: 1000 |
| Mature-IL-1β | Affinity biosciences | AF4006 | 1: 1000 |
| NLRP3 | Affinity biosciences | DF15549 | 1: 1000 |
| TRPV1 | Proteintech | 22686-1-AP | 1:1000 |
| GAPDH | Abcam, China | ab8245 | 1: 5000 |
| Goat Anti-Rabbit IgG H&L (HRP) | abcam, USA | ab97080 | 1: 5000 |
| Goat Anti-Mouse IgG H&L (HRP) | abcam, USA | ab97040 | 1: 5000 |
